# Long Pentraxin-3 Modulates the Angiogenic Activity of Fibroblast Growth Factor-2

**DOI:** 10.3389/fimmu.2018.02327

**Published:** 2018-10-08

**Authors:** Marco Presta, Eleonora Foglio, Ander Churruca Schuind, Roberto Ronca

**Affiliations:** Department of Molecular and Translational Medicine, School of Medicine, University of Brescia, Brescia, Italy

**Keywords:** angiogenesis, FGF, inflammation, PTX3, endothelium, cancer

## Abstract

Angiogenesis, the process of new blood vessel formation from pre-existing ones, plays a key role in various physiological and pathological conditions. Alteration of the angiogenic balance, consequent to the deranged production of angiogenic growth factors and/or natural angiogenic inhibitors, is responsible for angiogenesis-dependent diseases, including cancer. Fibroblast growth factor-2 (FGF2) represents the prototypic member of the FGF family, able to induce a complex “angiogenic phenotype” in endothelial cells *in vitro* and a potent neovascular response *in vivo* as the consequence of a tight cross talk between pro-inflammatory and angiogenic signals. The soluble pattern recognition receptor long pentraxin-3 (PTX3) is a member of the pentraxin family produced locally in response to inflammatory stimuli. Besides binding features related to its role in innate immunity, PTX3 interacts with FGF2 and other members of the FGF family via its N-terminal extension, thus inhibiting FGF-mediated angiogenic responses *in vitro* and *in vivo*. Accordingly, PTX3 inhibits the growth and vascularization of FGF-dependent tumors and FGF2-mediated smooth muscle cell proliferation and artery restenosis. Recently, the characterization of the molecular bases of FGF2/PTX3 interaction has allowed the identification of NSC12, the first low molecular weight pan-FGF trap able to inhibit FGF-dependent tumor growth and neovascularization. The aim of this review is to provide an overview of the impact of PTX3 and PTX3-derived molecules on the angiogenic, inflammatory, and tumorigenic activity of FGF2 and their potential implications for the development of more efficacious anti-FGF therapeutic agents to be used in those clinical settings in which FGFs play a pathogenic role.

## FGF2 as an angiogenic growth factor

Angiogenesis is a multistep process leading to the formation of new blood vessels from pre-existing ones. It occurs in different physiological and pathological settings, including embryonic development, wound repair, inflammation, and cancer. During the “angiogenic switch,” activated endothelial cells (ECs) degrade the basement membrane and start migrating (tip cells) and proliferating (stalk cells) to form EC sprouts that will originate vascular loops and capillary tubes with formation of tight junctions, deposition of a new basement membrane and pericyte recruitment (1, 2). The activation of ECs results from the balance between pro-angiogenic growth factors and anti-angiogenic players released by different perivascular cell types (2). A plethora of molecules have been described to regulate angiogenesis, including Fibroblast Growth Factor-2 (FGF2) that, together with FGF1, was first identified in the 1980s as a heparin-binding angiogenic factor (3, 4).

FGF2 exerts pleiotropic activities on target cells, including ECs, by interacting with cell surface heparan-sulfate proteoglycans (HSPGs) and high affinity tyrosine kinase receptors (FGFRs) (5). FGF2/FGFR interaction fosters the dimerization of the receptor and the autophosphorylation of its intracellular tyrosine kinase domain that, in turn, leads to the activation of complex signal transduction pathways (6).

Among the 23 members of the FGF family (5), FGF2 represents the most characterized and potent pro-angiogenic mediator *in vitro* and *in vivo* (7), even though a significant pro-angiogenic activity has been demonstrated also for FGF4 and FGF8 whereas it remains debated for other FGFs (including FGF5, FGF7, FGF9, FGF16, and FGF18) (8). *In vitro*, FGF2 induces EC proliferation and migration, promotes the production of proteases and expression of integrin and cadherin receptors (9).

*In vivo*, FGF2 stimulates the neovascularization process in different experimental models, including the chick embryo chorioallantoic membrane (CAM) (10), rabbit/mouse cornea (11, 12), zebrafish yolk membrane (ZFYM) (13), and murine subcutaneous Matrigel plug (14) assays. Conversely, loss of FGF signaling in ECs results in augmented vascular permeability and loss of vessel integrity (15). Notably, the pro-angiogenic function of FGF2 is mostly mediated by FGFR1, that represents the main FGFR expressed by activated ECs (9), and less frequently by FGFR2 (16), whereas FGFR3 and FGFR4 do not appear to be expressed in ECs.

Usually, the biological effect exerted by FGF2 on ECs is the consequence of a paracrine stimulation due to its release by inflammatory cells, stromal components or tumor cells, as well as by its mobilization from FGF-binding components that are present in the extracellular matrix (ECM) (6, 7, 17). Moreover, ECs can undergo autocrine or intracrine stimulation due to the self-production of FGF2 (18).

Finally, FGF2 stimulates lymphangiogenesis by direct and indirect (often vascular endothelial growth factor (VEGF)-C mediated) action on lymphatic endothelial cells (LECs), where it promotes proliferation, migration, and survival (19, 20). Recent observations have shown that FGF2 controls the glycolytic metabolism in ECs and LECs through a FGFR/MYC/Hexokinase 2-mediated pathway (21).

## FGF2-dependent angiogenesis and inflammation

Emerging evidence supports a role for inflammation in angiogenesis and suggests mutual dependency of the two processes in several physiological and pathological conditions (22, 23) due to common signaling pathways and mediators (24). During inflammatory reactions, the immune infiltrate may produce pro-inflammatory cytokines with pro-angiogenic properties, together with growth factors and proteases that contribute to the formation of new vascular structures (25, 26). The newly formed vasculature, in turn, sustains inflammation by facilitating the recruitment of inflammatory cells to the site of inflammation (27–29).

Noteworthy, elevated levels of FGF2 have been implicated in the pathogenesis of several diseases characterized by a deregulated angiogenic/inflammatory response, including cancer (7).

### Contribution of inflammatory cells in promoting FGF2-dependent angiogenesis

In response to phlogistic stimuli, inflammatory cells provide key cytokines and growth factors to the angiogenic vascular network and interact with endothelial surface adhesion molecules, affecting vascular permeability and inducing EC migration and proliferation (30–32). These cells can produce pro-angiogenic factors, including FGF2, that stimulate the proliferation and migration of hypoxic ECs, supporting a paracrine model for the modulation of EC growth at the inflammatory site. Thus, various cell types known to play a pivotal role in the initiation and progression of inflammation have been considered active players in angiogenesis (33–36). In this context, monocytes/macrophages (MCs/MPHs) (37, 38), T lymphocytes (34, 39) and mast cells (40) express FGF2 and their homing to inflammatory sites can impact the neovascular response associated to inflammation (41). In addition, platelet alpha granules represent a source of various angiogenic factors, including FGF2, that are released during physiological and pathological conditions and may contribute to angiogenic responses (42).

The involvement of MCs/MPHs in inflammatory angiogenesis has been reported in a variety of experimental settings (43). For instance, Polverini and colleagues found that activated MPHs and their cell culture media were able to induce neovascularization in the cornea assay, thus relating the angiogenic activity of macrophages with their secretome (44). MCs/MPHs are frequently associated with proliferating blood vessels where they accumulate and provide angiogenic growth factors, including FGF2, as is the case for coronary collaterals where the rapid vessel growth correlates with MC adhesion to the intima (45, 46).

Factors released by MCs/MPHs alter the tissue microenvironment, promoting EC migration, proliferation and new vessel formation (47, 48) and stimulate the migration of other accessory cells, in particular mast cells, able to potentiate the angiogenic response (29, 49). The early recruitment of MCs/MPHs (within 2–3days after implantation) precedes blood vessel formation in a FGF2-driven Matrigel plug angiogenesis assay (23). Accordingly, a significant reduction of the angiogenic response elicited by FGF2 and other angiogenic factors has been demonstrated following MC/MPH depletion induced by intraperitoneal pretreatment with clodronate liposomes (Clodrolip) (50, 51). Notably, MPHs may facilitate FGF signaling by producing heparinases and plasmin that degrade the ECM, thus disengaging ECM-bound FGF molecules that eventually will activate FGFRs in ECs, and create “guiding paths” for proliferating and migrating ECs (35, 43). Accordingly, long-term treatment with FGF2 stimulates ECM degradation by MCs/MPHs to facilitate the invasion of Tie2^+^ EC precursors and blood vessel formation in Matrigel implants (48).

The significant inhibition of the angiogenic response to FGF2 observed in neutropenic mice suggests that, similar to MCs/MPHs, neutrophils may play a key role in FGF2-mediated angiogenesis (32), most likely by producing additional pro-angiogenic cytokines and ECM-degrading proteases (52–54). On the other hand, neutrophil-derived elastase may favor FGF2 degradation, thus counteracting its angiogenic activity (55, 56).

The tissue density of mast cells is highly correlated with the extent of normal and pathologic angiogenesis (57). Mast cells are recruited by FGF2 (58) and, in turn, may release FGF2, as well as other pro-angiogenic factors, leading to EC activation (59, 60). Accordingly, mast cells and their isolated secretory granules induce an angiogenic response in the chick embryo CAM assay (61) that is inhibited by neutralizing anti-FGF2 antibodies (40).

More recently, it has been demonstrated that dendritic cells may sustain inflammatory neovascularization through the expression of a wide array of pro-angiogenic mediators (including FGF2, VEGF, and ETS-1) (62–66). In addition, similar to MCs, DCs may contribute to neovessel formation by differentiating into endothelial-like cells following treatment with FGF2, VEGF-A, and IGF-1 (67).

### FGF2 amplifies the EC response to inflammatory stimuli

ECs themselves may play important autocrine, intracrine, or paracrine roles in angiogenesis *via* FGF2 production (18), thus inducing a pro-angiogenic status in the endothelium that creates a favorable environment for vascular growth. FGF2 production and release from ECs can be triggered by inflammatory mediators such as IL-1β (68), nitric oxide (NO) (69), prostaglandin E2 (PGE_2_) (70), and IL-2 upon exposure of ECs to interferon-α (IFN-α) (71).

The observation that angiogenesis is accompanied by vasodilation prompted studies aimed to assess the involvement of vasodilators, like NO and PGE_2_, in the angiogenic activity of FGF2. Even though FGF2-induced angiogenesis can occur independently from NO production (72), elevation of NO levels in ECs increases their FGF2 production (72). Similarly, PGE_2_ exerts its pro-angiogenic action through paracrine activation of endothelial FGFR1 following mobilization of FGF2 sequestered in the ECM (70). Conversely, FGF2 and VEGF-A induce angiogenesis by increasing cyclooxygenase and PGE_2_ production (73, 74).

A transcriptome study on murine microvascular ECs demonstrated that FGF2-driven neovascularization induces a complex pro-inflammatory signature in the endothelium, with early upregulation of several inflammation-related genes (23). Even though also VEGF-A may upregulate the expression of inflammation-related genes in ECs (75–77), it remains unclear whether the two angiogenic mediators utilize distinct or common molecular pathways to exert their biological effects on ECs. Indeed, although an intimate cross-talk between FGF2 and VEGF-A during angiogenesis may exist (78), FGF2 appears to be responsible for the early induction of inflammation-related genes independently from VEGF expression, that represents a later event (23).

FGF2 amplifies the EC response to inflammatory stimuli by vasoactive effects and recruitment of a consistent inflammatory infiltrate. Besides inducing vasodilation of coronary arterioles through endothelial NO production (79), FGF2 increases vascular permeability *via* VEGF-A and protease upregulation (80). Moreover, FGF2 enhances the recruitment of MCs, T cells, and neutrophils (25) by increasing their adhesion and trans-endothelial migration *via* the upregulation/expression of the cell adhesion molecules ICAM-1 and VCAM-1 in ECs (81, 82).

Notably, studies from different groups suggest that FGF2 might have a context-dependent pro- or anti-inflammatory activity. While a rapid, transient exposure to FGF2 induces the upregulation of endothelial adhesion molecules that contribute to immune infiltrate recruitment, a prolonged exposure to FGF2 may result in a marked down-regulation of ICAM-1, VCAM-1, and E-selectin expression on ECs, accompanied by a strong reduction of adhesion and transmigration of monocytes, neutrophils and CD4^+^ T lymphocytes even in response to potent chemotactic factors (83–85). This biphasic effect of FGF2 might be one of the mechanisms utilized by cancer cells to escape from host immune reactions during the angiogenic stage of tumor development (86).

Finally, inflammation may also impair the angiogenic effects mediated by FGF2 *via* the production of molecules that sequester FGF2. For instance, the C-X-C chemokine platelet factor 4, a well-known inhibitor of angiogenesis released from alpha-granules of activated platelets, is able to bind FGF2, thus preventing FGFR activation and proliferation in ECs (87). A further, remarkable example is represented by long pentraxin-3 (PTX3), a member of the innate immunity with relevant functions in inflammatory responses and pathogen recognition, whose FGF2 antagonist activity will be discussed in details here below.

## PTX3/FGF interaction

### Biochemical interactions

The pentraxin family is a highly conserved group of pattern recognition glycoproteins implicated in innate immunity. PTX3, a prototypic member of the long pentraxin subfamily, is a 340 kDa octamer in which up to 92% of the amino acid sequence (each subunit being formed by 389 residues) is common between mouse and human proteins (88).

The roles played by PTX3 in innate immunity, wound healing/tissue remodeling, cardiovascular diseases, fertility, and infectious diseases span, among others, from opsonization to apoptotic cell clearance, extracellular matrix formation and FGF2 inhibition in tissue homeostasis (89). This functional variety is due to the complex structure of the protein. PTX3 has a unique N-terminal domain with non-redundant functions, whereas its C-terminal domain is common to all pentraxins and contains the “pentraxin signature” (89, 90). PTX3 contains an *N*-glycosylation site in Asn220 that contributes to the fine tuning of ligand binding (91).

The *N*-terminal domain of PTX3 binds FGF2 with high affinity (Kd ~ 30–300 nM) (92–94) and one octameric PTX3 molecule binds FGF2 in a 1 to 2 stoichiometric ratio (95). Using various biochemical approaches, the *N*-terminal amino acidic sequence 97–110 was recognized as responsible for FGF2 binding. Later, the acetylated pentapeptide Ac-ARPCA, corresponding to amino acids 100–104, was identified as the minimal sequence of PTX3 able to bind FGF2 (93, 96). Of note, PTX3 can interact *via* its *N*-terminal also with FGF8b, another member of the FGF family endowed with pro-angiogenic properties (97), and other family members, like FGF6, FGF10, and FGF17 (92).

**Figure 1:**
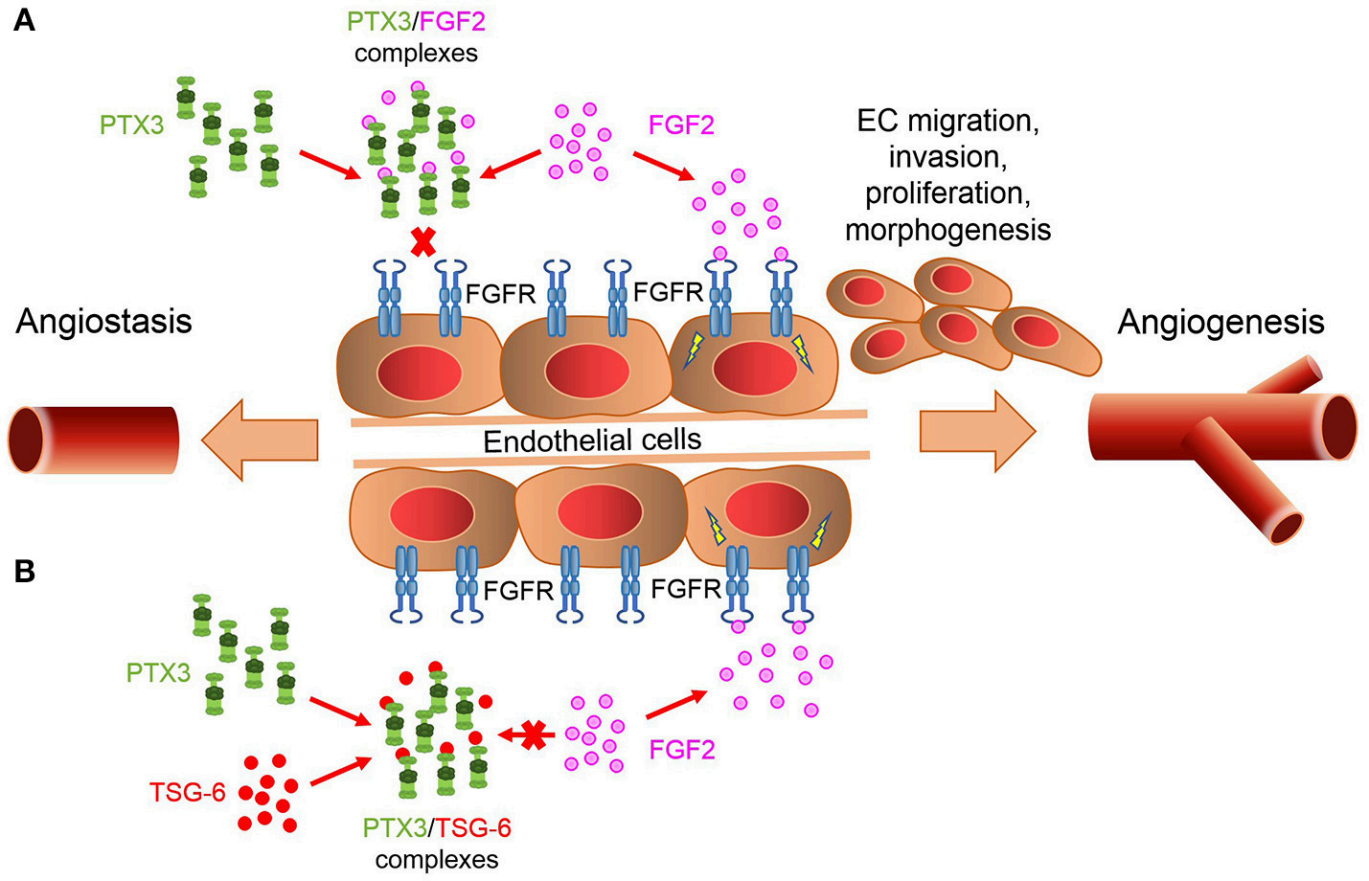
PTX3/TSG-6 interaction modulates FGF2-mediated angiogenesis. **(A)** PTX3 acts as a natural FGF trap, thus inhibiting FGF2/FGFR1 complex formation and angiogenesis. **(B)** TSG-6 binds PTX3 and prevents PTX3/FGF2 interaction. This abrogates the inhibitory effect exerted by PTX3 on FGF2 activity.

An important player in modulating PTX3/FGF2 interaction is represented by the tumor necrosis factor-stimulated gene-6 (TSG-6) protein. TSG-6 is expressed in inflamed and neovascularization sites by lymphocytes, smooth muscle cells, and ECs in response to inflammatory stimuli (98). TSG-6 binds PTX3 and other ECM components, like hyaluronic acid and the heavy chains of inter-α-inhibitor, thus allowing the formation of intricate molecular webs in the ECM (99, 100). TSG-6 binds the PTX3 *N*-terminus and prevents its interaction with FGF2, thus reverting the inhibition exerted by PTX3 on FGF2 activity. This may provide a mechanism to control angiogenesis in those inflammatory conditions characterized by the co-expression of TSG-6 and PTX3, in which the relative levels of these proteins may act as a biological rheostat to fine-tune the angiogenic activity of FGF2 (101) (Figure 1).

### Biological implications

**Figure 2:**
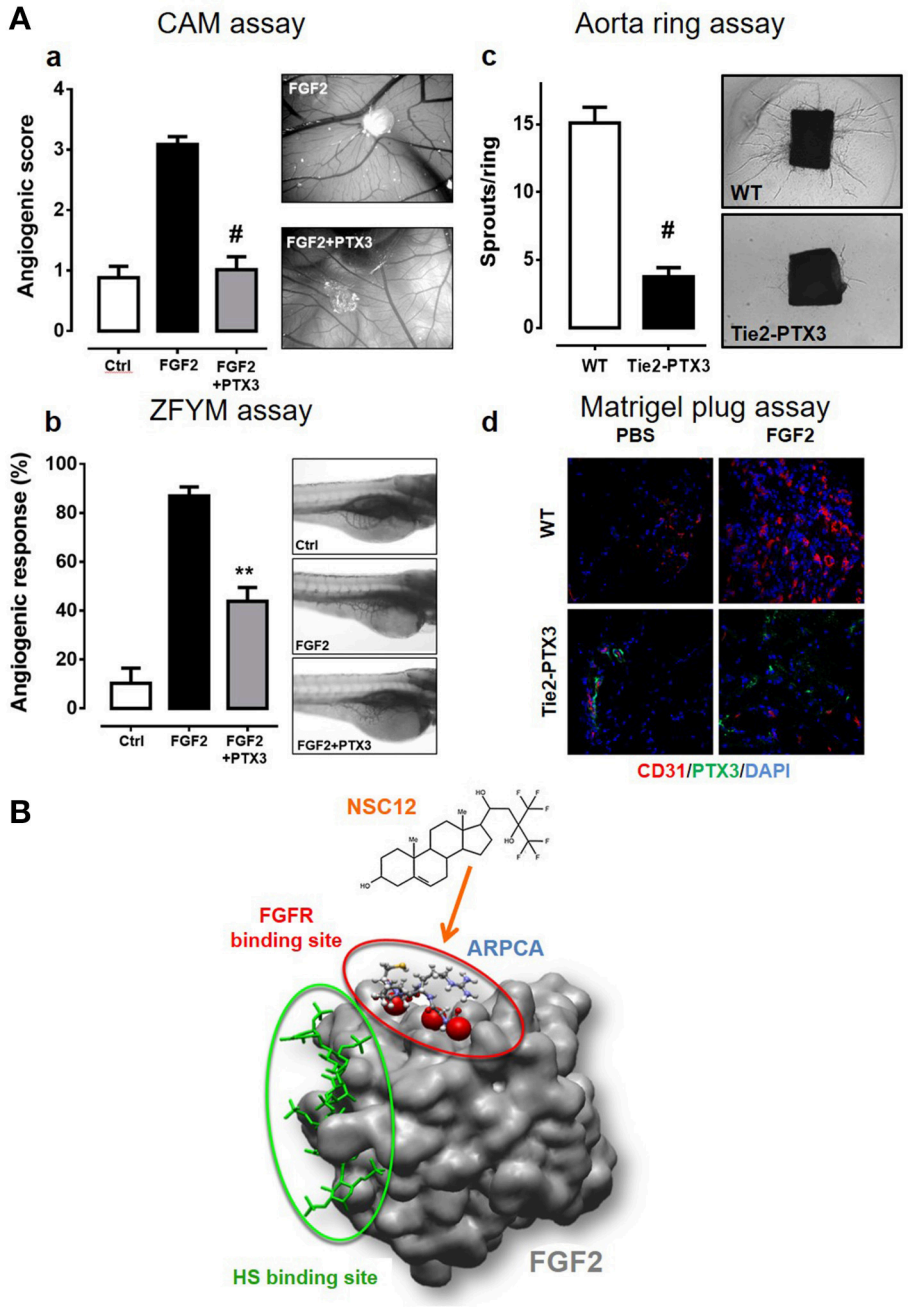
PTX3 inhibits the angiogenic activity of FGF2. **(A)** When tested in different angiogenesis models, a molar excess of purified PTX3 protein **(a,b)** or its transgenic endothelial overexpression **(c,d)** inhibits the neovascular response triggered by an optimal dose of recombinant FGF2 [see references (13), (94), (106) for details] ^**^p < 0.01; ^#^*p* < 0.001. **(B)** The PTX3-derived pentapeptide ARPCA (ball and stick representation) interacts with the FGFR-binding domain of FGF2 (red circle) without affecting its heparin-binding region (green circle). A similar mechanism of action is hypothesized for the FGF trap small molecule NSC12.

PTX3/FGF2 interaction prevents the formation of the biologically active HSPG/FGF2/FGFR ternary complex, thus inhibiting FGF2-dependent EC activation and angiogenesis (94, 102). *In vitro* experiments demonstrated that the *N*-terminal domain of PTX3 and the PTX3-derived ARPCA pentapeptide impair the proliferation/activation of ECs in response to FGF2 but not to VEGF-A, thus confirming the specificity of the effect (94, 96). *In vivo*, PTX3 significantly hampers the angiogenic response triggered by alginate beads adsorbed with FGF2 and implanted on the chick embryo CAM (Figures 2Aa) (96). Similar results were obtained in a zebrafish/tumor xenograft model (103) where the angiogenic response to FGF2-overexpressing tumor cells was strongly impaired by the co-injection of PTX3 or ARPCA (Figures 2Ab) (96). Accordingly, overexpression of PTX3 by tumor cells of different origin (including melanoma, prostate, and breast cancer cells) causes a significant inhibition of tumor-associated neovascularization and FGF-dependent tumor growth (92, 104, 105).

The effect of PTX3 overexpression on ECs was assessed in a transgenic mouse model where the human *Ptx3* gene was under the control of endothelial-specific Tie2 promoter [TgN(Tie2-hPTX3) mice] (106). When isolated from the lung of TgN(Tie2-hPTX3) animals, PTX3-overexpressing ECs showed a reduced capacity to respond to exogenous FGF2 in terms of cell proliferation and 3D-sprouting when compared to ECs isolated from wild type animals (106). This was accompanied by a significant reduction of endothelial FGFR1 activation/phosphorylation following stimulation with FGF2. In agreement with these observations, the overexpression of PTX3 by the endothelium of transgenic animals caused a significant inhibition of the angiogenic response triggered by FGF2 in an *ex vivo* murine aorta ring assay and *in vivo* when TgN(Tie2-hPTX3) mice were tested in a Matrigel plug assay (Figures 2Acd). No inhibitory effect was observed when VEGF-A was used an angiogenic stimulus, thus confirming that the anti-angiogenic activity of PTX3 was directly mediated by the impairment of the FGF2/FGFR1 axis. As a consequence of the anti-FGF2/anti-angiogenic activity of PTX3, FGF2-dependent syngeneic tumor grafts of different origin were characterized by impaired FGFR1 activation and reduced CD31^+^ vascularization and tumor growth when injected in TgN(Tie2-hPTX3) mice (106). Notably, the TRAMP-C2 prostate adenocarcinoma cell grafts generated in TgN(Tie2-hPTX3) mice were characterized also by a significant decrease of the mast cell infiltrate into the lesion (58). These data, in keeping with previous observations about the capacity of mast cells to respond chemotactically to FGF2, provide evidence about a relationship among FGF2-dependent mast cell recruitment, angiogenesis, and tumor growth in prostate adenocarcinoma, all hampered by PTX3.

Moreover, when considering the role of FGF2 in the formation and maintenance of lymphatic vessels (19, 20), it is possible to hypothesize that PTX3 may inhibit FGF2-mediated lymphangiogenesis and its associated events, including tumor metastatic dissemination (107). Further experiments are required to assess this hypothesis.

The anti-angiogenic/anti-tumor activity of PTX3 was not restricted to FGF2. Indeed, due to its capacity to bind FGF8b, PTX3 prevents the interaction of this FGF family member with FGFR1 and blocks FGF8b-induced EC proliferation and chemotaxis *in vitro* and angiogenesis *in vivo*, causing a significant inhibition of tumor growth and vascularization when transduced in androgen-regulated Shionogi 115 mouse breast tumor cells (97) that express both FGF2 and FGF8b following stimulation with dihydrotestosterone (105).

PTX3 binds extracellular matrix component of the vessel wall, including collagen and fibrinogen, thus affecting platelet aggregation (108), In addition, it can bind activated circulating platelets and dampen their proinflammatory and prothrombotic action (109). It will be of interest to assess whether such interactions may result in the sequestration of platelet-released FGF2, with a consequent modulation of its bioavailability and biological activity in different thrombosis-prone conditions, including tissue ischemia, wound healing, atherosclerosis, and cancer.

### Therapeutic implications

When considering its FGF2 antagonist activity, PTX3 might be regarded as a potential therapeutic agent in those pathological settings in which FGF2 exerts a driving role. Endovascular injection of adeno-associated virus harboring the *PTX3* cDNA was used to block FGF2-mediated intimal thickening after balloon injury in the rat carotid artery (110) whereas its retroviral/lentiviral transduction has been exploited to inhibit FGF activity in different tumor models (102). However, due to its size (340 kDa), complex quaternary structure (homo-octamer), and proteinaceous nature, any pharmacological application of PTX3 protein appears unrealistic unless functional “shuttles” can be identified for this “cargo.” One possibility for a direct therapeutic exploitation of the PTX3 protein has been shown by using “tumor targeting” Tie2^+^ monocytes (TEMs) (111) derived from the bone marrow of TgN(Tie2-hPTX3) mice (106). In this experimental model, PTX3-expressing TEMs were able to efficiently deliver the PTX3 protein to the tumor site in a syngeneic FGF2-dependent model of prostate cancer, causing a significant reduction of the growth of the tumor grafts (106).

In order to set the basis for the development of novel PTX3-derived FGF2 antagonists with potential therapeutic implications, the PTX3-derived pentapeptide ARPCA was characterized in preclinical models of FGF-dependent angiogenesis and cancer. Acetylated ARPCA appears to bind the FGF2 protein in a region responsible for its interaction with the D2-D3 linker and D3 domain of FGFR1 (Figure 2B) and inhibits the angiogenic activity exerted by FGF2/FGF8, as well as the FGF-dependent growth of prostate and androgen-dependent breast tumors (96, 105). More recently, based on the analysis of ARPCA/FGF2 interaction, molecular modeling and small molecule library screening, a PTX3-derived 480 Da compound (named NSC12, Figure 2B) was identified as the first small molecule to function as a pan FGF2 trap (106, 112). Indeed, NSC12 binds and impairs the biological activity of all the canonical FGF family members and displays significant anti-angiogenic activities *in vitro, ex vivo* and *in vivo* in a series of FGF2-dependent angiogenesis assays, with no effect on VEGF-dependent EC activation (106). In addition, *in vivo* experiments performed on FGF-dependent models of prostate and lung cancer confirmed the capacity of NSC12 to inhibit FGFR1 activation and to reduce tumor growth and tumor-associated angiogenesis (26, 74). The non-aminoacidic structure of NSC12 makes this molecule a promising candidate for the development of more efficacious anti-FGF therapeutic agents to be used in clinical settings.

It must be pointed out that, at variance with tyrosine kinase FGFR inhibitors, FGF trapping following PTX3 overexpression in transgenic mice, as well as long-term NSC12 administration (106) or treatment with the FGFR-derived decoy molecule FP-1039 (113), are all devoid of significant toxic effects. This appears to be in contrast with the alterations of vascular integrity observed after systemic overexpression of soluble FGFRs in transgenic mice (15) and calls for further experiments aimed at assessing the therapeutic window of FGF trapping agents.

In conclusion, FGF2/PTX3 interaction may exert a deep impact on the angiogenesis process during inflammation and tumor growth. The balance among these interactors and other FGF and/or PTX3 binding molecules (e.g., TSG-6, ECM components and HSPGs) may further modulate neovessel formation under different physio/pathological conditions. A better understanding of these interactions may provide valuable insights into the pathogenesis of angiogenesis-dependent diseases and will set the basis for the development of novel therapeutic agents.

## Author contributions

All the authors contributed to the writing of the manuscript, MP and RR revised the final version.

### Conflict of interest statement

The authors declare that the research was conducted in the absence of any commercial or financial relationships that could be construed as a potential conflict of interest.

## References

[1] Carmeliet P. Mechanisms of angiogenesis and arteriogenesis. Nat Med. (2000) 6:389–95. 10.1038/74651 10742145

[2] Ronca R Benkheil M Mitola S Struyf S Liekens S. Tumor angiogenesis revisited: regulators and clinical implications. Med Res Rev. (2017) 37:1231–74. 10.1002/med.21452 28643862

[3] Shing Y Folkman J Sullivan R Butterfield C Murray J Klagsbrun M. Heparin affinity: purification of a tumor-derived capillary endothelial cell growth factor. Science (1984) 223:1296–9. 6199844 10.1126/science.6199844

[4] Maciag T Mehlman T Friesel R Schreiber AB. Heparin binds endothelial cell growth factor, the principal endothelial cell mitogen in bovine brain. Science (1984) 225:932–5. 6382607 10.1126/science.6382607

[5] Katoh M Nakagama H. FGF receptors: cancer biology and therapeutics. Med Res Rev. (2014) 34:280–300. 10.1002/med.21288 23696246

[6] Giacomini A Chiodelli P Matarazzo S Rusnati M Presta M Ronca R. Blocking the FGF/FGFR system as a “two-compartment” antiangiogenic/antitumor approach in cancer therapy. Pharmacol Res. (2016) 107:172–85. 10.1016/j.phrs.2016.03.024 27013279

[7] Presta M Dell'Era P Mitola S Moroni E Ronca R Rusnati M. Fibroblast growth factor/fibroblast growth factor receptor system in angiogenesis. Cytokine Growth Factor Rev. (2005) 16:159–78. 10.1016/j.cytogfr.2005.01.004 15863032

[8] Ronca R Giacomini A Rusnati M Presta M. The potential of fibroblast growth factor/fibroblast growth factor receptor signaling as a therapeutic target in tumor angiogenesis. Expert Opin Ther Targets (2015) 19:1361–77. 10.1517/14728222.2015.1062475 26125971

[9] Javerzat S Auguste P Bikfalvi A. The role of fibroblast growth factors in vascular development. Trends Mol Med. (2002) 8:483–9. 10.1016/S1471-4914(02)02394-8 12383771

[10] Ribatti D Vacca A Roncali L Dammacco F. The chick embryo chorioallantoic membrane as a model for *in vivo* research on anti-angiogenesis. Curr Pharm Biotechnol. (2000) 1:73–82. 10.2174/1389201003379040 11467363

[11] Herbert JM Laplace MC Maffrand JP. Effect of heparin on the angiogenic potency of basic and acidic fibroblast growth factors in the rabbit cornea assay. Int J Tissue React. (1988) 10:133–9. 2465281

[12] Seghezzi G Patel S Ren CJ Gualandris A Pintucci G Robbins ES. Fibroblast growth factor-2 (FGF-2) induces vascular endothelial growth factor (VEGF) expression in the endothelial cells of forming capillaries: an autocrine mechanism contributing to angiogenesis. J Cell Biol. (1998) 141:1659–73. 9647657 10.1083/jcb.141.7.1659 PMC2132998

[13] Nicoli S De Sena G Presta M. Fibroblast growth factor 2-induced angiogenesis in zebrafish: the zebrafish yolk membrane (ZFYM) angiogenesis assay. J Cell Mol Med. (2009) 13:2061–8. 10.1111/j.1582-4934.2008.00432.x 18657228 PMC6512384

[14] Coltrini D Di Salle E Ronca R Belleri M Testini C Presta M. Matrigel plug assay: evaluation of the angiogenic response by reverse transcription-quantitative PCR. Angiogenesis (2013) 16:469–77. 10.1007/s10456-012-9324-7 23143707

[15] Murakami M Nguyen LT Zhuang ZW Moodie KL Carmeliet P Stan RV. The FGF system has a key role in regulating vascular integrity. J Clin Invest. (2008) 118:3355–66. 10.1172/JCI35298 18776942 PMC2528913

[16] Dell'Era P Belleri M Stabile H Massardi ML Ribatti D Presta M. Paracrine and autocrine effects of fibroblast growth factor-4 in endothelial cells. Oncogene (2001) 20:2655–63. 10.1038/sj.onc.1204368 11420677

[17] Ronca R Van Ginderachter JA Turtoi A. Paracrine interactions of cancer-associated fibroblasts, macrophages and endothelial cells: tumor allies and foes. Curr Opin Oncol. (2018) 30:45–53. 10.1097/CCO.0000000000000420 29084000

[18] Gualandris A Rusnati M Belleri M Nelli EE Bastaki M Molinari-Tosatti MP. Basic fibroblast growth factor overexpression in endothelial cells: an autocrine mechanism for angiogenesis and angioproliferative diseases. Cell Growth Differ. (1996) 7:147–60. 8822198

[19] Chang LK Garcia-Cardena G Farnebo F Fannon M Chen EJ Butterfield C. Dose-dependent response of FGF-2 for lymphangiogenesis. Proc Natl Acad Sci USA. (2004) 101:11658–63. 10.1073/pnas.0404272101 15289610 PMC511009

[20] Shin JW Min M Larrieu-Lahargue F Canron X Kunstfeld R Nguyen L. Prox1 promotes lineage-specific expression of fibroblast growth factor (FGF) receptor-3 in lymphatic endothelium: a role for FGF signaling in lymphangiogenesis. Mol Biol Cell (2006) 17:576–84. 10.1091/mbc.e05-04-0368 16291864 PMC1356570

[21] Yu P Wilhelm K Dubrac A Tung JK Alves TC Fang JS. FGF-dependent metabolic control of vascular development. Nature (2017) 545:224–8. 10.1038/nature22322 28467822 PMC5427179

[22] Carmeliet P Jain RK. Angiogenesis in cancer and other diseases. Nature (2000) 407:249–57. 10.1038/35025220 11001068

[23] Andres G Leali D Mitola S Coltrini D Camozzi M Corsini M. A pro-inflammatory signature mediates FGF2-induced angiogenesis. J Cell Mol Med. (2009) 13:2083–108. 10.1111/j.1582-4934.2008.00415.x 18624773 PMC6512373

[24] Szade A Grochot-Przeczek A Florczyk U Jozkowicz A Dulak J. Cellular and molecular mechanisms of inflammation-induced angiogenesis. IUBMB Life (2015) 67:145–59. 10.1002/iub.1358 25899846

[25] Presta M Andres G Leali D Dell'era P Ronca R. Inflammatory cells and chemokines sustain FGF2-induced angiogenesis. Eur Cytokine Netw. (2009) 20:39–50. 10.1684/ecn.2009.0155 19541589

[26] Zijlstra A Seandel M Kupriyanova TA Partridge JJ Madsen MA Hahn-Dantona EA. Proangiogenic role of neutrophil-like inflammatory heterophils during neovascularization induced by growth factors and human tumor cells. Blood (2006) 107:317–27. 10.1182/blood-2005-04-1458 16174763 PMC1895349

[27] Aplin AC Gelati M Fogel E Carnevale E Nicosia RF. Angiopoietin-1 and vascular endothelial growth factor induce expression of inflammatory cytokines before angiogenesis. Physiol Genomics (2006) 27:20–8. 10.1152/physiolgenomics.00048.2006 17018690

[28] Danese S Dejana E Fiocchi C. Immune regulation by microvascular endothelial cells: directing innate and adaptive immunity, coagulation, and inflammation. J Immunol. (2007) 178:6017–22. 10.4049/jimmunol.178.10.6017 17475823

[29] Ribatti D Crivellato E. Immune cells and angiogenesis. J Cell Mol Med. (2009) 13:2822–33. 10.1111/j.1582-4934.2009.00810.x 19538473 PMC4498938

[30] Bussolino F Mantovani A Persico G. Molecular mechanisms of blood vessel formation. Trends Biochem Sci. (1997) 22:251–6. 9255066 10.1016/s0968-0004(97)01074-8

[31] Hanahan D Folkman J. Patterns and emerging mechanisms of the angiogenic switch during tumorigenesis. Cell (1996) 86:353–64. 8756718 10.1016/s0092-8674(00)80108-7

[32] Shaw JP Chuang N Yee H Shamamian P. Polymorphonuclear neutrophils promote rFGF-2-induced angiogenesis *in vivo*. J Surg Res. (2003) 109:37–42. 10.1016/S0022-4804(02)00020-3 12591233

[33] Mor F Quintana FJ Cohen IR. Angiogenesis-inflammation cross-talk: vascular endothelial growth factor is secreted by activated T cells and induces Th1 polarization. J Immunol. (2004) 172:4618–23. 10.4049/jimmunol.172.7.4618 15034080

[34] Blotnick S Peoples GE Freeman MR Eberlein TJ Klagsbrun M. T lymphocytes synthesize and export heparin-binding epidermal growth factor-like growth factor and basic fibroblast growth factor, mitogens for vascular cells and fibroblasts: differential production and release by CD4+ and CD8+ T cells. Proc Natl Acad Sci USA. (1994) 91:2890–94. 7909156 10.1073/pnas.91.8.2890 PMC43479

[35] Moldovan NI Goldschmidt-Clermont PJ Parker-Thornburg J Shapiro SD Kolattukudy PE. Contribution of monocytes/macrophages to compensatory neovascularization: the drilling of metalloelastase-positive tunnels in ischemic myocardium. Circ Res. (2000) 87:378–84. 10.1161/01.RES.87.5.378 10969035

[36] Sunderkotter C Steinbrink K Goebeler M Bhardwaj R Sorg C. Macrophages and angiogenesis. J Leukoc Biol. (1994) 55:410–22. 7509844 10.1002/jlb.55.3.410

[37] Kuwabara K Ogawa S Matsumoto M Koga S Clauss M Pinsky DJ. Hypoxia-mediated induction of acidic/basic fibroblast growth factor and platelet-derived growth factor in mononuclear phagocytes stimulates growth of hypoxic endothelial cells. Proc Natl Acad Sci USA. (1995) 92:4606–10. 7538678 10.1073/pnas.92.10.4606 PMC41993

[38] Baird A Mormede P Bohlen P. Immunoreactive fibroblast growth factor in cells of peritoneal exudate suggests its identity with macrophage-derived growth factor. Biochem Biophys Res Commun. (1985) 126:358–64. 3970698 10.1016/0006-291x(85)90614-x

[39] Peoples GE Blotnick S Takahashi K Freeman MR Klagsbrun M Eberlein TJ. T lymphocytes that infiltrate tumors and atherosclerotic plaques produce heparin-binding epidermal growth factor-like growth factor and basic fibroblast growth factor: a potential pathologic role. Proc Natl Acad Sci USA. (1995) 92:6547–51. 7604030 10.1073/pnas.92.14.6547 PMC41555

[40] Ribatti D Crivellato E Candussio L Vacca A Nico B Benagiano V. Angiogenic activity of rat mast cells in the chick embryo chorioallantoic membrane is down-regulated by treatment with recombinant human alpha-2a interferon and partly mediated by fibroblast growth factor-2. Haematologica (2002) 87:465–71. 12010658

[41] Anderton SM Wraith DC. Selection and fine-tuning of the autoimmune T-cell repertoire. Nat Rev Immunol. (2002) 2:487–98. 10.1038/nri842 12094223

[42] Bertrand-Duchesne MP Grenier D Gagnon G. Epidermal growth factor released from platelet-rich plasma promotes endothelial cell proliferation *in vitro*. J Periodontal Res. (2010) 45:87–93. 10.1111/j.1600-0765.2009.01205.x 19602111

[43] Sunderkotter C Goebeler M Schulze-Osthoff K Bhardwaj R Sorg C. Macrophage-derived angiogenesis factors. Pharmacol Ther. (1991) 51:195–216. 1784630 10.1016/0163-7258(91)90077-y

[44] Polverini PJ Cotran PS Gimbrone MA Jr. Unanue ER. Activated macrophages induce vascular proliferation. Nature (1977) 269:804–6. 927505 10.1038/269804a0

[45] Schaper J Konig R Franz D Schaper W. The endothelial surface of growing coronary collateral arteries. Intimal margination and diapedesis of monocytes. A combined SEM and TEM study. Virchows Arch A Pathol Anat Histol. (1976) 370:193–205. 821235 10.1007/BF00427580

[46] Arras M Ito WD Scholz D Winkler B Schaper J Schaper W. Monocyte activation in angiogenesis and collateral growth in the rabbit hindlimb. J Clin Invest. (1998) 101:40–50. 10.1172/JCI119877 9421464 PMC508538

[47] Crosby JR Kaminski WE Schatteman G Martin PJ Raines EW Seifert RA. Endothelial cells of hematopoietic origin make a significant contribution to adult blood vessel formation. Circ Res. (2000) 87:728–30. 10.1161/01.RES.87.9.728 11055974

[48] Anghelina M Krishnan P Moldovan L Moldovan NI. Monocytes/macrophages cooperate with progenitor cells during neovascularization and tissue repair: conversion of cell columns into fibrovascular bundles. Am J Pathol. (2006) 168:529–41. 10.2353/ajpath.2006.050255 16436667 PMC1606496

[49] Gruber BL Marchese MJ Kew R. Angiogenic factors stimulate mast-cell migration. Blood (1995) 86:2488–93. 7545457

[50] Sakurai E Anand A Ambati BK van Rooijen N Ambati J. Macrophage depletion inhibits experimental choroidal neovascularization. Invest Ophthalmol Vis Sci. (2003) 44:3578–85. 10.1167/iovs.03-0097 12882810

[51] Nakao S Kuwano T Tsutsumi-Miyahara C Ueda S Kimura YN Hamano S. Infiltration of COX-2-expressing macrophages is a prerequisite for IL-1 beta-induced neovascularization and tumor growth. J Clin Invest. (2005) 115:2979–91. 10.1172/JCI23298 16239969 PMC1257532

[52] Tazzyman S Lewis CE Murdoch C. Neutrophils: key mediators of tumour angiogenesis. Int J Exp Pathol. (2009) 90:222–31. 10.1111/j.1365-2613.2009.00641.x 19563607 PMC2697547

[53] McCourt M Wang JH Sookhai S Redmond HP. Proinflammatory mediators stimulate neutrophil-directed angiogenesis. Arch Surg. (1999) 134:1325–31; discussion 31–2. 10593330 10.1001/archsurg.134.12.1325

[54] Ericson SG Zhao Y Gao H Miller KL Gibson LF Lynch JP Interleukin-6 production by human neutrophils after Fc-receptor cross-linking or exposure to granulocyte colony-stimulating factor. Blood (1998) 91:2099–107. 9490696

[55] Ai S Cheng XW Inoue A Nakamura K Okumura K Iguchi A. Angiogenic activity of bFGF and VEGF suppressed by proteolytic cleavage by neutrophil elastase. Biochem Biophys Res Commun. (2007) 364:395–401. 10.1016/j.bbrc.2007.10.027 17950695

[56] Tecchio C Cassatella MA. Neutrophil-derived cytokines involved in physiological and pathological angiogenesis. Chem Immunol Allergy (2014) 99:123–37. 10.1159/000353358 24217606

[57] Meininger CJ. Mast cells and tumor-associated angiogenesis. Chem Immunol. (1995) 62:239–57. 7546284

[58] Ronca R Tamma R Coltrini D Ruggieri S Presta M Ribatti D. Fibroblast growth factor modulates mast cell recruitment in a murine model of prostate cancer. Oncotarget (2017) 8:82583–92. 10.18632/oncotarget.19773 29137286 PMC5669912

[59] Coussens LM Raymond WW Bergers G Laig-Webster M Behrendtsen O Werb Z. Inflammatory mast cells up-regulate angiogenesis during squamous epithelial carcinogenesis. Genes Dev. (1999) 13:1382–97. 10364156 10.1101/gad.13.11.1382 PMC316772

[60] Meininger CJ Zetter BR. Mast cells and angiogenesis. Semin Cancer Biol. (1992) 3:73–9. 1378312

[61] Mignatti P Morimoto T Rifkin DB. Basic fibroblast growth factor, a protein devoid of secretory signal sequence, is released by cells via a pathway independent of the endoplasmic reticulum-Golgi complex. J Cell Physiol. (1992) 151:81–93. 10.1002/jcp.1041510113 1560052

[62] Conejo-Garcia JR Benencia F Courreges MC Kang E Mohamed-Hadley A Buckanovich RJ. Tumor-infiltrating dendritic cell precursors recruited by a beta-defensin contribute to vasculogenesis under the influence of Vegf-A. Nat Med. (2004) 10:950–8. 10.1038/nm1097 15334073

[63] Coukos G Benencia F Buckanovich RJ Conejo-Garcia JR. The role of dendritic cell precursors in tumour vasculogenesis. Br J Cancer (2005) 92:1182–7. 10.1038/sj.bjc.6602476 15785750 PMC2361965

[64] Bosisio D Ronca R Salvi V Presta M Sozzani S. Dendritic cells in inflammatory angiogenesis and lymphangiogenesis. Curr Opin Immunol. (2018) 53:180–6. 10.1016/j.coi.2018.05.011 29879585

[65] Presta M Chiodelli P Giacomini A Rusnati M Ronca R. Fibroblast growth factors (FGFs) in cancer: FGF traps as a new therapeutic approach. Pharmacol Ther. (2017) 179:171–87. 10.1016/j.pharmthera.2017.05.013 28564583

[66] Riboldi E Musso T Moroni E Urbinati C Bernasconi S Rusnati M. Cutting edge: proangiogenic properties of alternatively activated dendritic cells. J Immunol. (2005) 175:2788–92. 10.4049/jimmunol.175.5.2788 16116163

[67] Fernandez Pujol B Lucibello FC Zuzarte M Lutjens P Muller R Havemann K. Dendritic cells derived from peripheral monocytes express endothelial markers and in the presence of angiogenic growth factors differentiate into endothelial-like cells. Eur J Cell Biol. (2001) 80:99–110. 10.1078/0171-9335-00136 11211940

[68] Lee HT Lee JG Na M Kay EP. FGF-2 induced by interleukin-1 beta through the action of phosphatidylinositol 3-kinase mediates endothelial mesenchymal transformation in corneal endothelial cells. J Biol Chem. (2004) 279:32325–32. 10.1074/jbc.M405208200 15173165

[69] Walford G Loscalzo J. Nitric oxide in vascular biology. J Thromb Haemost. (2003) 1:2112–8. 10.1046/j.1538-7836.2003.00345.x 14521592

[70] Finetti F Solito R Morbidelli L Giachetti A Ziche M Donnini S. Prostaglandin E2 regulates angiogenesis via activation of fibroblast growth factor receptor-1. J Biol Chem. (2008) 283:2139–46. 10.1074/jbc.M703090200 18042549

[71] Cozzolino F Torcia M Lucibello M Morbidelli L Ziche M Platt J. Interferon-alpha and interleukin 2 synergistically enhance basic fibroblast growth factor synthesis and induce release, promoting endothelial cell growth. J Clin Invest. (1993) 91:2504–12. 7685771 10.1172/JCI116486 PMC443311

[72] Ziche M Morbidelli L Choudhuri R Zhang HT Donnini S Granger HJ Nitric oxide synthase lies downstream from vascular endothelial growth factor-induced but not basic fibroblast growth factor-induced angiogenesis. J Clin Invest. (1997) 99:2625–34. 10.1172/JCI119451 9169492 PMC508108

[73] Salcedo R Zhang X Young HA Michael N Wasserman K Ma WH. Angiogenic effects of prostaglandin E2 are mediated by up-regulation of CXCR4 on human microvascular endothelial cells. Blood (2003) 102:1966–77. 10.1182/blood-2002-11-3400 12791666

[74] Hernandez GL Volpert OV Iniguez MA Lorenzo E Martinez-Martinez S Grau R. Selective inhibition of vascular endothelial growth factor-mediated angiogenesis by cyclosporin A: roles of the nuclear factor of activated T cells and cyclooxygenase 2. J Exp Med. (2001) 193:607–20. 10.1084/jem.193.5.607 11238591 PMC2193389

[75] Reinders ME Sho M Izawa A Wang P Mukhopadhyay D Koss KE. Proinflammatory functions of vascular endothelial growth factor in alloimmunity. J Clin Invest. (2003) 112:1655–65. 10.1172/JCI17712 14660742 PMC281640

[76] Kim I Moon SO Kim SH Kim HJ Koh YS Koh GY. Vascular endothelial growth factor expression of intercellular adhesion molecule 1 (ICAM-1), vascular cell adhesion molecule 1 (VCAM-1), and E-selectin through nuclear factor-kappa B activation in endothelial cells. J Biol Chem. (2001) 276:7614–20. 10.1074/jbc.M009705200 11108718

[77] Abe M Sato Y. cDNA microarray analysis of the gene expression profile of VEGF-activated human umbilical vein endothelial cells. Angiogenesis (2001) 4:289–98. 10.1023/A:1016018617152 12197474

[78] Jih YJ Lien WH Tsai WC Yang GW Li C Wu LW. Distinct regulation of genes by bFGF and VEGF-A in endothelial cells. Angiogenesis (2001) 4:313–21. 10.1023/A:1016080321956 12197476

[79] Tiefenbacher CP Chilian WM. Basic fibroblast growth factor and heparin influence coronary arteriolar tone by causing endothelium-dependent dilation. Cardiovasc Res. (1997) 34:411–7. 9205556 10.1016/s0008-6363(97)00029-1

[80] Reuss B Dono R Unsicker K. Functions of fibroblast growth factor (FGF)-2 and FGF-5 in astroglial differentiation and blood-brain barrier permeability: evidence from mouse mutants. J Neurosci. (2003) 23:6404–12. 10.1523/JNEUROSCI.23-16-06404.2003 12878680 PMC6740627

[81] Zittermann SI Issekutz AC. Endothelial growth factors VEGF and bFGF differentially enhance monocyte and neutrophil recruitment to inflammation. J Leukoc Biol. (2006) 80:247–57. 10.1189/jlb.1205718 16818728

[82] Zittermann SI Issekutz AC. Basic fibroblast growth factor (bFGF, FGF-2) potentiates leukocyte recruitment to inflammation by enhancing endothelial adhesion molecule expression. Am J Pathol. (2006) 168:835–46. 10.2353/ajpath.2006.050479 16507899 PMC1606526

[83] Zhang H Issekutz AC. Growth factor regulation of neutrophil-endothelial cell interactions. J Leukoc Biol. (2001) 70:225–32. 10.1189/jlb.70.2.225 11493614

[84] Zhang H Issekutz AC. Down-modulation of monocyte transendothelial migration and endothelial adhesion molecule expression by fibroblast growth factor: reversal by the anti-angiogenic agent SU6668. Am J Pathol. (2002) 160:2219–30. 10.1016/S0002-9440(10)61169-8 12057924 PMC1850845

[85] Griffioen AW Damen CA Martinotti S Blijham GH Groenewegen G. Endothelial intercellular adhesion molecule-1 expression is suppressed in human malignancies: the role of angiogenic factors. Cancer Res. (1996) 56:1111–17. 8640769

[86] Kitayama J Nagawa H Yasuhara H Tsuno N Kimura W Shibata Y. Suppressive effect of basic fibroblast growth factor on transendothelial emigration of CD4(+) T-lymphocyte. Cancer Res. (1994) 54:4729–33. 7520360

[87] Perollet C Han ZC Savona C Caen JP Bikfalvi A. Platelet factor 4 modulates fibroblast growth factor 2 (FGF-2) activity and inhibits FGF-2 dimerization. Blood (1998) 91:3289–99. 9558385

[88] Garlanda C Bottazzi B Bastone A Mantovani A. Pentraxins at the crossroads between innate immunity, inflammation, matrix deposition, and female fertility. Annu Rev Immunol. (2005) 23:337–66. 10.1146/annurev.immunol.23.021704.115756 15771574

[89] Presta M Camozzi M Salvatori G Rusnati M. Role of the soluble pattern recognition receptor PTX3 in vascular biology. J Cell Mol Med. (2007) 11:723–38. 10.1111/j.1582-4934.2007.00061.x 17760835 PMC3823252

[90] Breviario F d'Aniello EM Golay J Peri G Bottazzi B Bairoch A. Interleukin-1-inducible genes in endothelial cells. Cloning of a new gene related to C-reactive protein and serum amyloid P component. J Biol Chem. (1992) 267:22190–7. 1429570

[91] Inforzato A Peri G Doni A Garlanda C Mantovani A Bastone A. Structure and function of the long pentraxin PTX3 glycosidic moiety: fine-tuning of the interaction with C1q and complement activation. Biochemistry (2006) 45:11540–51. 10.1021/bi0607453 16981714

[92] Ronca R Alessi P Coltrini D Di Salle E Giacomini A Leali D. Long pentraxin-3 as an epithelial-stromal fibroblast growth factor-targeting inhibitor in prostate cancer. J Pathol. (2013) 230:228–38. 10.1002/path.4181 23424081

[93] Camozzi M Rusnati M Bugatti A Bottazzi B Mantovani A Bastone A. Identification of an antiangiogenic FGF2-binding site in the N terminus of the soluble pattern recognition receptor PTX3. J Biol Chem. (2006) 281:22605–13. 10.1074/jbc.M601023200 16769728

[94] Rusnati M Camozzi M Moroni E Bottazzi B Peri G Indraccolo S. Selective recognition of fibroblast growth factor-2 by the long pentraxin PTX3 inhibits angiogenesis. Blood (2004) 104:92–9. 10.1182/blood-2003-10-3433 15031207

[95] Inforzato A Baldock C Jowitt TA Holmes DF Lindstedt R Marcellini M. The angiogenic inhibitor long pentraxin PTX3 forms an asymmetric octamer with two binding sites for FGF2. J Biol Chem. (2010) 285:17681–92. 10.1074/jbc.M109.085639 20363749 PMC2878532

[96] Leali D Bianchi R Bugatti A Nicoli S Mitola S Ragona L. Fibroblast growth factor 2-antagonist activity of a long-pentraxin 3-derived anti-angiogenic pentapeptide. J Cell Mol Med. (2010) 14:2109–21. 10.1111/j.1582-4934.2009.00855.x 19627396 PMC3823002

[97] Leali D Alessi P Coltrini D Ronca R Corsini M Nardo G. Long pentraxin-3 inhibits FGF8b-dependent angiogenesis and growth of steroid hormone-regulated tumors. Mol Cancer Ther. (2011) 10:1600–10. 10.1158/1535-7163.MCT-11-0286 21764903

[98] Bayliss MT Howat SL Dudhia J Murphy JM Barry FP Edwards JC. Up-regulation and differential expression of the hyaluronan-binding protein TSG-6 in cartilage and synovium in rheumatoid arthritis and osteoarthritis. Osteoarthritis Cartilage (2001) 9:42–8. 10.1053/joca.2000.0348 11178946

[99] Kohda D Morton CJ Parkar AA Hatanaka H Inagaki FM Campbell ID. Solution structure of the link module: a hyaluronan-binding domain involved in extracellular matrix stability and cell migration. Cell (1996) 86:767–75. 8797823 10.1016/s0092-8674(00)80151-8

[100] Baranova NS Nileback E Haller FM Briggs DC Svedhem S Day AJ et als. The inflammation-associated protein TSG-6 cross-links hyaluronan via hyaluronan-induced TSG-6 oligomers. J Biol Chem. (2011) 286:25675–86. 10.1074/jbc.M111.247395 21596748 PMC3138277

[101] Leali D Inforzato A Ronca R Bianchi R Belleri M Coltrini D. Long pentraxin 3/tumor necrosis factor-stimulated gene-6 interaction: a biological rheostat for fibroblast growth factor 2-mediated angiogenesis. Arterioscler Thromb Vasc Biol. (2012) 32:696–703. 10.1161/ATVBAHA.111.243998 22267482 PMC3551298

[102] Giacomini A Ghedini GC Presta M Ronca R. Long pentraxin 3: a novel multifaceted player in cancer. BBA Rev Cancer (2018) 1869:53–63. 10.1016/j.bbcan.2017.11.004 29175552

[103] Nicoli S Presta M. The zebrafish/tumor xenograft angiogenesis assay. Nat Protoc. (2007) 2:2918–23. 10.1038/nprot.2007.412 18007628

[104] Ronca R Di Salle E Giacomini A Leali D Alessi P Coltrini D. Long pentraxin-3 inhibits epithelial-mesenchymal transition in melanoma cells. Mol Cancer Ther. (2013) 12:2760–71. 10.1158/1535-7163.MCT-13-0487 24130051

[105] Giacomini A Matarazzo S Pagano K Ragona L Rezzola S Corsini M. A long pentraxin-3-derived pentapeptide for the therapy of FGF8b-driven steroid hormone-regulated cancers. Oncotarget (2015) 6:13790–802. 10.18632/oncotarget.3831 25912421 PMC4537050

[106] Ronca R Giacomini A Di Salle E Coltrini D Pagano K Ragona L. Long-Pentraxin 3 Derivative as a small-molecule FGF trap for cancer therapy. Cancer Cell (2015) 28:225–39. 10.1016/j.ccell.2015.07.002 26267536

[107] Cao R Ji H Feng N Zhang Y Yang X Andersson P. Collaborative interplay between FGF-2 and VEGF-C promotes lymphangiogenesis and metastasis. Proc Natl Acad Sci USA. (2012) 109:15894–9. 10.1073/pnas.1208324109 22967508 PMC3465417

[108] Bonacina F Barbieri SS Cutuli L Amadio P Doni A Sironi M. Vascular pentraxin 3 controls arterial thrombosis by targeting collagen and fibrinogen induced platelets aggregation. Biochim Biophys Acta (2016) 1862:1182–90. 10.1016/j.bbadis.2016.03.007 26976330 PMC4856734

[109] Maugeri N Rovere-Querini P Slavich M Coppi G Doni A Bottazzi B. Early and transient release of leukocyte pentraxin 3 during acute myocardial infarction. J Immunol. (2011) 187:970–9. 10.4049/jimmunol.1100261 21677138

[110] Camozzi M Zacchigna S Rusnati M Coltrini D Ramirez-Correa G Bottazzi B. Pentraxin 3 inhibits fibroblast growth factor 2-dependent activation of smooth muscle cells *in vitro* and neointima formation *in vivo*. Arterioscler Thromb Vasc Biol. (2005) 25:1837–42. 10.1161/01.ATV.0000177807.54959.7d 16020751

[111] De Palma M Murdoch C Venneri MA Naldini L Lewis CE Tie2-expressing monocytes: regulation of tumor angiogenesis and therapeutic implications. Trends Immunol. (2007) 28:519–24. 10.1016/j.it.2007.09.004 17981504

[112] Castelli R Giacomini A Anselmi M Bozza N Vacondio F Rivara S. Synthesis, structural elucidation, and biological evaluation of NSC12, an orally available fibroblast growth factor (FGF) ligand trap for the treatment of FGF-dependent lung tumors. J Med Chem. (2016) 59:4651–63. 10.1021/acs.jmedchem.5b02021 27138345

[113] Harding TC Long L Palencia S Zhang H Sadra A Hestir K. Blockade of nonhormonal fibroblast growth factors by FP-1039 inhibits growth of multiple types of cancer. Sci Transl Med. (2013) 5:178ra39. 10.1126/scitranslmed.3005414 23536011

